# Whole-exome analysis of foetal autopsy tissue reveals a frameshift mutation in *OBSL1*, consistent with a diagnosis of 3-M Syndrome

**DOI:** 10.1186/1471-2164-16-S1-S12

**Published:** 2015-01-15

**Authors:** Christian R Marshall, Sandra A Farrell, Donna Cushing, Tara Paton, Tracy L Stockley, Dimitri J Stavropoulos, Peter N Ray, Michael Szego, Lynette Lau, Sergio L Pereira, Ronald D Cohn, Richard F Wintle, Adel M Abuzenadah, Muhammad Abu-Elmagd, Stephen W Scherer

**Affiliations:** 1 The Centre for Applied Genomics and Program in Genetics and Genome Biology, The Hospital for Sick Children, Toronto, Ontario, M5G 0A4, Canada; 2 McLaughlin Centre and Department of Molecular Genetics, the University of Toronto, Ontario, M5S 2J7, Canada; 3 Genetics, Trillium Health Partners, Credit Valley Hospital, Mississauga, Ontario, L5M 2N1, Canada; 4 Department of Paediatric Laboratory Medicine, The Hospital for Sick Children, Toronto, Ontario, M5G 1X8, Canada; 5 Joint Centre for Bioethics and Department of Family and Community Medicine, the University of Toronto, Ontario, M5T 1P8, Canada; 6 Division of Clinical and Metabolic Genetics, The Hospital for Sick Children, Toronto, Ontario, Canada; 7 Centre of Excellence in Genomic Medicine Research, King Abdulaziz University, 80216 Jeddah 21589, Kingdom of Saudi Arabia; 8 KACST Technology Innovation Center in Personalized Medicine, King Abdulaziz University, Jeddah, Kingdom of Saudi Arabia; 9 Zoology Department, Faculty of Science, Minia University, Minia, Egypt

**Keywords:** exome sequencing, frameshift mutation, *OBSL1*, skeletal dysplasia, 3-M Syndrome

## Abstract

**Background:**

We report a consanguineous couple that has experienced three consecutive pregnancy losses following the foetal ultrasound finding of short limbs. Post-termination examination revealed no skeletal dysplasia, but some subtle proximal limb shortening in two foetuses, and a spectrum of mildly dysmorphic features. Karyotype was normal in all three foetuses (46, XX) and comparative genomic hybridization microarray analysis detected no pathogenic copy number variants.

**Results:**

Whole-exome sequencing and genome-wide homozygosity mapping revealed a previously reported frameshift mutation in the *OBSL1* gene (c.1273insA p.T425nfsX40), consistent with a diagnosis of 3-M Syndrome 2 (OMIM #612921), which had not been anticipated from the clinical findings.

**Conclusions:**

Our study provides novel insight into the early clinical manifestations of this form of 3-M syndrome, and demonstrates the utility of whole exome sequencing as a tool for prenatal diagnosis in particular when there is a family history suggestive of a recurrent set of clinical symptoms.

## Introduction

3-M syndrome is an autosomal recessive disorder characterized by severe pre- and postnatal growth retardation and a characteristic facial appearance, but notably with normal intellect. Typical features include a relatively large head proportionate to the limbs, triangular face, hypoplastic midface, full eyebrows, fleshy nose tip, and long philtrum [1]. Other features can include prominent heels, loose joints, short broad neck, square shoulders, and hyperlordosis. Mutations in three genes are known to cause 3-M syndrome: *CUL7* (type 1; OMIM #273750), *OBSL1* (type 2; OMIM #612921), and *CCDC8* (type 3; OMIM #614205), with roughly 65% of 3-M syndrome mutations described in *CUL7*, and 30% in *OBSL1*. Functional analysis suggests that proteins encoded by these three genes interact in a pathway involved in insulin-like growth factor signalling [2].

**Figure 1:**
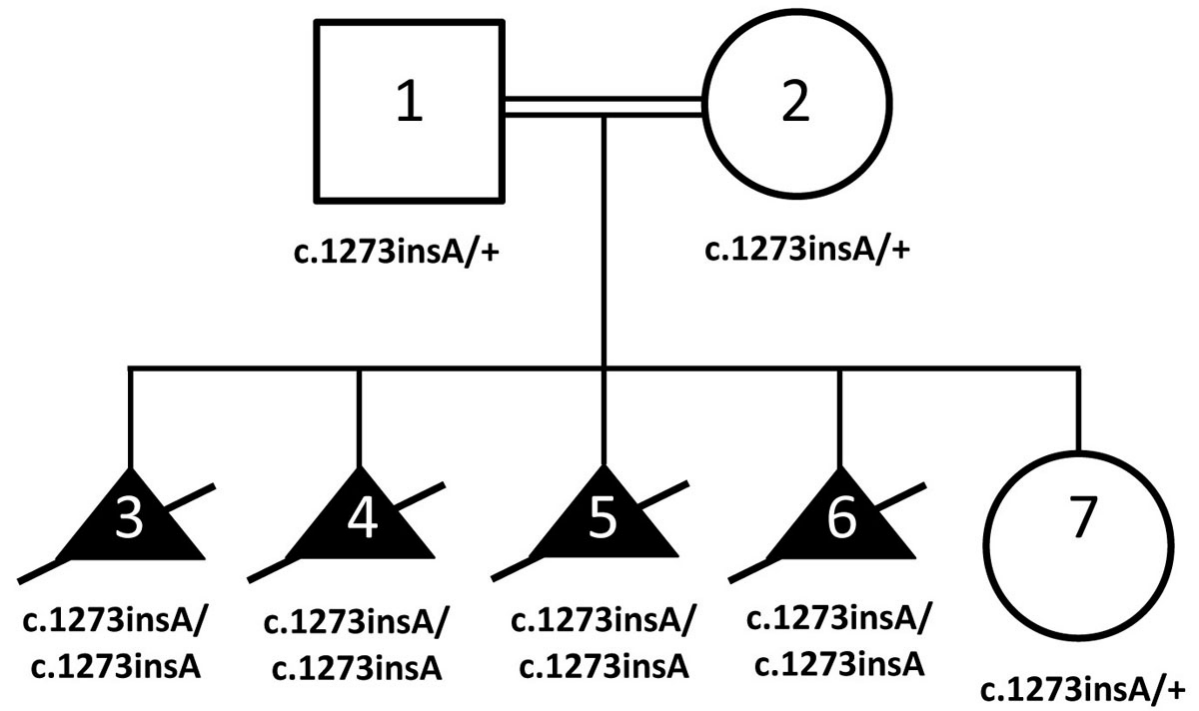
Pedigree with segregation of *OBSL1* c.1273insA variant (p.T425nfsX40) in four elective terminations. Individuals 1-5 were genotyped with high-resolution SNP microarray analysis with subsequent homozygosity mapping. Individuals 3 and 4 underwent whole exome sequencing. Individuals 6 and 7 underwent targeted variant testing in the clinical diagnostic laboratory.

We report a first cousin marriage consanguineous couple that experienced three consecutive pregnancy losses following the foetal ultrasound finding of short limbs (Figure 1). Clinical features are summarized in Table 1. The couple was seen in their first pregnancy initially because of an increased nuchal translucency (NT) measurement (5.2 mm at 13.5 weeks gestation). Amniocentesis showed normal female chromosomes (46, XX). Ultrasound examination at 19 weeks gestation (as estimated by last menstrual period (LMP) date and previous ultrasound) demonstrated normal foetal anatomy, but the femoral and humeral lengths lagged by two weeks. Bone morphology appeared normal, as was a foetal echocardiogram. Repeat ultrasounds at 25 weeks and 28 weeks showed foetal long bones lagging by four weeks and five weeks, respectively. The pregnancy was ended. Radiographs taken post-termination showed no evidence of a skeletal dysplasia. This fetus was not examined by a geneticist and nor was there an autopsy.

**Table 1:** Phenotypic Features of 3-M syndrome in three affected foetuses.

CATEGORY	SUBCATEGORY	FEATURES	3^a^	4^b^	5^b^
Growth	Height	Short stature	+	+	+
	
	Weight	Low birth weight	NA	NA	NA
	
	Other	Intrauterine growth retardation	+	+	+
		
		Postnatal growth retardation	NA	NA	NA

Head and Neck	Head	Frontal bossing	ND	+	+
		
		Increased relative head circumference	ND	+	+
	
	Face	Triangular face	ND	+	+
		
		Pointed, prominent chin	ND	+	+
		
		Hypoplastic midface	ND	+	+
		
		Long philtrum	ND	ND	+
	
	Eyes	Full eyebrows	NA	NA	NA
	
	Nose	Fleshy, upturned nose	ND	ND	ND
		
		Low nasal bridge	ND	+	+
		
		Depressed nasal root	ND	ND	ND
		
		Anteverted nares	ND	ND	+
	
	Mouth	Full lips	NA	NA	NA
	
	Neck	Short neck	NA	NA	NA

Respiratory		Neonatal respiratory distress	NA	NA	NA

Chest	External Features	Short, wide, flat thorax	NA	NA	NA
		
		Pectus excavatum	NA	NA	NA
	
	Ribs, Sternum, Clavicles and Scapulae	High, square shoulders	NA	NA	NA
		
		Rib hypoplasia	NA	NA	NA
		
		Winged scapulae	NA	NA	NA

Abdomen	External Features	Enlarged abdomen	NA	NA	NA

Genitourinary	External Genitalia (Male)	Hypospadias	NA	NA	NA
		
		Small testes	NA	NA	NA

Skeletal	General	Delayed bone age	+	+	+
		
		Joint hypermobility	NA	NA	NA
		
		Joint dislocation	NA	NA	NA
	
	Skull	Dolichocephaly	ND	ND	ND
	
	Spine	Tall vertebral bodies	ND	ND	ND
		
		Hyperlordosis	NA	NA	NA
	
	Pelvis	Hip dislocation	NA	NA	NA
		
		Small pelvis	NA	NA	NA
	
	Limbs	Long, slender tubular bones	NA	+	+
	
	Hands	Short fifth fingers	NA	NA	NA
		
		Clinodactyly	NA	NA	NA
	
	Feet	Prominent heels	NA	+	+
		
		Pes planus	NA	NA	NA

Skin, Nails, Hair	Hair	Full eyebrows	NA	NA	NA

Neurologic	Central Nervous System	Normal intelligence	NA	NA	NA
		
		Spina bifida occulta	NA	NA	NA

^a^ultrasound and radiograph; ^b^formal autopsy performed; NA (Not applicable); ND (Not done or tested)

In the couple’s second pregnancy, the NT measured 2.8 mm at 13 weeks gestation. Foetal ultrasound at 18 weeks gestation, by LMP and previous ultrasound, showed foetal long bones lagging by two weeks. Bone morphology was again normal. Ultrasound at 22 weeks showed foetal long bones lagging by three weeks. The pregnancy was ended. Microarray analysis detected no pathogenic variants. On autopsy, the foetus was mildly dysmorphic with increased bitemporal diameter, slightly upslanting palpebral fissures, prominent and mildly proptotic eyes, low set ears, mild nuchal edema, a proportionately large head, proximal limb shortening, and prominent heel pads. Autopsy was otherwise unremarkable, including microscopy of bones. Postmortem radiographs showed no evidence of a skeletal dysplasia.

In the third pregnancy, the NT measured 3.5 mm at 11 weeks gestation. Ultrasound at 20 weeks gestation, by LMP and previous ultrasound, showed foetal long bones lagging by two weeks. The pregnancy was ended. On autopsy, the foetus showed a triangular face associated with hypoplasia of maxillary bones and mandibles, synophrys, prominent eyes, and long philtrum. Proximal limbs were shortened. Karyotype showed normal female chromosomes (46, XX).

Given the couple’s history of consanguinity and no definitive clinical explanation for the medical symptoms, DNA samples from all three foetuses and parents were tested by whole-exome sequence analysis as part of a clinical research study. Assuming an autosomal recessive model of inheritance, exome sequencing was attempted with the goal of uncovering previously unknown mutations in coding regions of genes. This approach has been shown particularly effective when combined with genome-wide microarray genotyping or conventional linkage analysis to identify regions of homozygosity in consanguineous pedigrees [3-7]. We report here the discovery of a previously known frameshift mutation in *OBSL1*, providing a diagnosis of 3-M Syndrome 2, which would explain the foetal long bone ultrasound findings. This diagnosis was not anticipated based on the prenatal clinical observations alone. The counselling and diagnostic issues raised by this discovery are discussed.

## Materials and Methods

Subjects were consented for research study at The Hospital for Sick Children (Toronto, Canada; REB File No.: 1000007909).

### Karyotyping, Genome Wide SNP genotyping, homozygosity mapping, and Copy Number Variation (CNV) analysis

DNA samples from all family members were isolated from whole blood or autopsy material. Karyotypes were performed on a clinical basis using standard techniques. Samples were genotyped for 2,443,177 markers using the Infinium HumanOmni2.5-quad v1.0 BeadChip (Illumina Inc., San Diego, CA, USA) according to the manufacturer’s protocol. Briefly, 200 ng of DNA (4 μL at 50 μg/μL) was independently amplified, labeled, and hybridized to BeadChip microarrays then scanned with default settings using an Illumina iScan. Analysis and intra-chip normalization of resulting image files was performed using Illumina’s GenomeStudio Genotyping Module software v.2011 with default parameters. Genotype calls were generated using the vendor-provided genotype cluster definitions file (HumanOmni2.5-4v1_H.egt, generated using HapMap project DNA samples) with a Gencall cutoff of 0.15. The CNVPartition v.3.1.6 module in GenomeStudio was used for CNV analysis and homozygosity mapping. Potential CNVs were compared to control data in the *Database of Genomic Variants* (http://projects.tcag.ca/variation) [8,9] and also examined for recurrence in all three affected foetuses. Approximately 700,000 markers were also analyzed for shared regions of homozygosity shared by the three affected offspring using HomozygosityMapper [10].

### Whole Exome Sequencing

Paired-end (75 + 35 nucleotide) exome sequencing was performed on samples from two affected foetuses (TCAG0322-003 and TCAG0322-004) on a SOLiD 5500*xl* (Life Technologies, Carlsbad, CA, USA) with target enrichment using the SureSelect 50 Mb Human All Exon capture kit V3 (Agilent Technologies, Santa Clara, CA, USA). Briefly, 3 μg of genomic DNA was sonicated to approximately 200 bp using a Covaris-S2 (Covaris Inc., Woburn, MA, USA). Fragmented DNA was end-repaired and purified with Agencourt Ampure XP beads (Beckman Coulter, Inc.). Fragments were size selected to ~200 bp through electrophoresis on a 2% agarose E-gel (Life Technologies), and the recovered DNA amplified using SureSelect pre-capture primers and Herculase II Fusion Enzyme (Agilent) with conditions of 72°C for 20 minutes, 95°C for 5 minutes, followed by 10 cycles of 95°C for 15 seconds, 54°C for 45 seconds, 70°C for 60 seconds and a subsequent final extension of 70°C for five minutes. Hybridization with biotinylated RNA baits corresponding to target regions was carried out using 500 ng of prepared library for 24 hours at 65°C, and captured DNA recovered with streptavidin coated magnetic beads. The recovered library was amplified and barcode sequencing tags incorporated by amplification with Herculase II Fusion Enzyme and SureSelect barcoding primers under conditions of 95°C for 5 minutes, then 95°C for 15 seconds, 54°C for 45 seconds, 70°C for 60 seconds for 9 cycles, and a subsequent final extension of 70°C for 5 minutes. Equimolar quantities of six barcoded exome libraries were pooled for preparation of templated beads by emulsion PCR using the Life Technology EZ Bead System. Beads were loaded on six lanes of a 5500 flowchip for paired-end sequencing as recommended by the manufacturer.

### Read Mapping, Variant Calling and Annotation

Paired end reads for both samples were combined and mapped to the reference human genome (UCSC hg19) using BFAST (http://bfast.sourceforge.net) [11]. Indel and SNP calls were made using GATK version1.0.5506 and recommended parameters [12]. Base quality score recalibration optimized to SOLiD 5500xl was used to remove reference bias. All no-call reads were removed from the analysis, and if a reference base was inserted the base quality was set to zero and the inserted reference nucleotide set to “N”. Variant recalibration with external datasets including HapMap, 1000 genomes, and dbSNP 129 was performed before using Beagle [13], implemented in GATK, to refine genotypes. Variants were filtered using cutoffs from GATK results of QD (Quality by Depth) >5 and SB (Strand Bias) <0.01 to minimize false positive detection. Variant calls were designated as novel if they did not appear in the external datasets listed above. Variants were annotated using SIFT 4.0.3 [14] to determine if the substitution was predicted to be deleterious to protein function.

### Sequence Validation

The *OBSL1* variant was validated using standard Sanger sequencing. A 453 bp region of *OBSL1* containing the variant was PCR-amplified using the primers OBSL1-F (5’- GCACAGCATTCTCTCCTTCC -3’) and OBSL1-R (5’- CCAGAGCCCTCTCTTGTCTC - 3’) using 50n g of genomic DNA. The 25 μl reaction consisted of 22 μl PCR master mix (7.8 μl water, 6.3 μl 5M betaine, 3.1 μl 10x PCR buffer (Life Technologies), 1.5 μl 25mM MgCl2, 3.1 μl 2mM dNTP mix, 0.2 μl AmpliTaq DNA polymerase (Life Technologies)), 1μl each primer (50 ng/μl) and 1ul DNA (50 ng/μl). Cycling conditions were 95°C (10 min) followed by 36 cycles of 95°C (15 sec) – 60°C (30 sec) – 72°C (1 min).

## Results

Homozygosity mapping using high-resolution genotyping revealed two regions that reached significance: one of 9.7 Mb at chromosome 2q34-q35, and one of 35.7 Mb at chromosome 8q13.2-q22.3 present for all three affected foetuses (Additional File 1, Table S1 and Figure S1). These regions were both heterozygous in the biological parents. Our CNV analysis did not reveal any potentially pathogenic alterations for any of the three foetuses (data not shown).

Exome sequencing yielded a total of 19,838 genic variants for further investigation (Additional File 1, Table S2). We prioritized rare (<1% allele frequency) or novel variants that were either loss of function (frameshift, splice mutations, stop mutations) or missense variants that were predicted to be damaging and that resided within the regions of shared homozygosity described above. At total of 13 variants (Additional File 1, Table S3) were detected that fit these criteria involving nine genes (*SPAG16*, *MARCH4*, *GPBAR1*, *USP37*, *ANKZF1*, *SPEG*, *OBSL1*, *SLCO5A1*, *DCAF13*).

The most interesting candidate was a homozygous insertion variant in the gene *OBSL1* (OMIM #610991) on chromosome 2 (c.1273insA), resulting in a frameshift and premature stop (p.T425nfsX40). This frameshift variant of *OBSL1* has been previously observed in patients with 3-M syndrome [15,16] and in those studies was detected in cases with a similar ancestry to the family in this study. Moreover, the finding of a recessive loss of function mutation in OBSL1 called our attention to 3-M syndrome being similar to the phenotype observed in the foetuses. Despite the majority of 3M core clinical features not manifesting completely until later in life (Table 1), the skeletal phenotype observed in the foetuses ultimately made a prenatal diagnosis of 3-M syndrome plausible. The variant was validated by Sanger sequencing, and demonstrated segregation consistent with an autosomal recessive model of disease inheritance (i.e., heterozygous in both parents, homozygous in all three foetuses).

At the time of our initial research findings we learned that the female research subject was pregnant for the fourth time. Clinical confirmation of the research finding though approved molecular diagnostic gene testing was denied by the provincial (Ontario) Ministry of Health that oversees clinical testing since in this jurisdiction it will not authorise testing on material from a deceased individual. Through consultation with attending physicians and ethicists genetic counselling was eventually offered based on the research result and a sample was taken for clinical testing in the diagnostic laboratory at The Hospital for Sick Children. The clinical testing revealed a homozygous variant genotype, consistent with an affected child. This pregnancy was eventually ended. The fifth pregnancy was similarly tested in the clinical diagnostic laboratory, indicating the foetus was a carrier of the *OBSL1* mutation. This was consistent with the normal developmental pattern subsequently observed.

## Discussion

Prenatal diagnosis of 3-M syndrome based on ultrasound is findings is unreliable, given that intrauterine growth retardation has many causes and is not specific to this syndrome. Additionally, although growth retardation of long bones *in utero* has been reported [17], this finding is not always seen in children with 3-M syndrome. As well, the degree of long bone growth lag of these fetuses was relatively mild and might have been missed had there not been an increased NT measurement to cause careful scrutiny of the fetal ultrasound results in the first pregnancy. Although in this family, at autopsy, the second and third foetuses were noted to have physical findings seen in 3-M syndrome (large head, heel pads, prenatal growth retardation, triangular face, hypoplastic midface and long philtrum) these findings were not specific enough by themselves to consider a differential diagnosis of 3-M syndrome.

Recently, exome sequencing has been used to reveal a mutation in the *CUL7* gene in a young adult with a previously undiagnosed growth disorder [18]. This observation echoes our finding that the spectrum of growth disorders represented by the genes of the *OBSL1*/*CUL7*/*CCDC8* growth factor signalling pathway might be broader than previously appreciated [19]. While *CUL7* and *OBSL1* account for ~94% of known 3-M syndrome cases [1], the remaining 6% (at least partly accounted for by *CCDC8* mutations) of undiagnosed cases suggests that additional genes and perhaps regulatory (non-coding) mutations in known genes are yet to be implicated.

Individual research results normally need to meet four threshold conditions prior to disclosure to research subjects. The results should have significant health implications for the participant, be actionable, and the test needs to be analytically valid. Furthermore, during the informed consent process the possibility of the return of individual results needs to be addressed and agreed upon by the research participant. In this study, we could not achieve the threshold of analytic validity, which in this case meant the test had to be performed in a licenced and accredited laboratory as required by legislation. Although our results were validated by whole exome and Sanger sequencing, respectively, the initial genetic experiment was performed in a research laboratory. To achieve analytic validity, in most jurisdictions, a validated genetic test in a clinical laboratory would have been required. We also had the added complication of provincial rules regarding testing of a sample from a deceased foetus. Given that the female research subject was pregnant for the fourth time and had already received abnormal ultrasound results our multidisciplinary team decided to disclose our research findings to the family through a hospital genetic counsellor. Our reasoning was that by providing this information to the research subject, she could then opt to undergo clinical prenatal testing for 3-M syndrome, an analytically valid test, which would not otherwise be clearly indicated at an early gestation by ultrasound findings. We were concerned that if we waited to achieve analytic validity, the research subject would likely have terminated her fourth pregnancy before the clinical diagnostic results were available. By returning the results back to the research subject, she was able to have a clinical test performed and make a more informed decision about her pregnancy. When the fourth foetus also was found to have the homozygous mutations, the pregnancy was ended at an earlier gestation than had occurred in the previously affected pregnancies, with the clear understanding that the foetus was affected.

Several regions of the world including some Middle Eastern gulf countries such as Saudi Arabia can have rates of consanguinity that can exceed 50% of unions necessitating implementation of clinical genetic programs [20-22]. Such high consanguinity has been shown to have serious medical impact on the populations with a high incidence of congenital malformations and mental retardation within the consanguineous families. This case illustrates how next generation sequencing, either whole exome or genome sequencing, can have power to establish prenatal diagnoses in foetuses that present with clinical phenotypes that do not provide enough specific details to establish a diagnosis based on ultrasound and clinical findings alone. In turn, knowledge of the mutation has provided the family with the opportunity to make informed decisions about pre-implantation testing/*in vitro* fertilization and/or to perform directed mutation analysis from foetal tissue via CVS or amniocentesis. The application of exome or whole genome sequencing in pediatric and adult disorders can raise different issues ranging from incidental or secondary findings [23], implications for genetic counselling and clinical management [24] to the direction of new treatment options [25]. We believe that whole genome sequencing thus represents a particular powerful diagnostic tool for families in areas with high incidence of consanguinity and has the potential to provide highly specific information to families and health care providers that will impact medical management beyond the current available technologies and diagnostic measures.

## Competing Interests

The authors declare that they have no competing interests.

## Supplementary Material

Additional file 1 Supplementary Material Click here for file

## Declarations

Publication fees were paid by the Centre of Excellence in Genomic Medicine Research of King Abdulaziz University.

This article has been published as part of *BMC Genomics* Volume 16 Supplement 1, 2015: Selected articles from the 2nd International Genomic Medical Conference (IGMC 2013): Genomics. The full contents of the supplement are available online at http://www.biomedcentral.com/bmcgenomics/supplements/16/S1
