# Supplementary material for: Whole-exome analysis of foetal autopsy tissue reveals a frameshift mutation in OBSL1, consistent with a diagnosis of 3-M Syndrome
Source: BMC Genomics. 2015 Jan 15;16(Suppl 1):S12. doi: 10.1186/1471-2164-16-S1-S12 (PMC4315153; doi:10.1186/1471-2164-16-S1-S12)
Supplement: Additional file 1 — Supplementary Material [file 1471-2164-16-S1-S12-S1.docx]

**Additional File 1: Supplementary Material**

**Table S1: Blocks of shared homozygosity between the three affected individuals**

| **chr** | **Start (hg19)** | **End (hg19)** | **SNP start** | **SNP end** | **Number of SNPs** | **Length (Mb)** |
| --- | --- | --- | --- | --- | --- | --- |
| 2 | 210,818,971 | 220,505,265 | rs16843977 | rs684428 | 91 | 9.69 |
| 8 | 70,206,163 | 105,884,484 | rs1462030 | rs7004837 | 163 | 35.7 |

**Table S2: Summary of Variant Filtering**

| **Category** | **Filtered variants** |
| --- | --- |
| Total autosomal SNV calls | 43,837 |
| Exonic SNVs | 19,838 |
| Nonsynonymous SNVs | 9,720 |
| Novel or Rare Nonsynonymous (NS) SNVs | 1,840 |
| Novel or Rare NS SNVs in regions of shared homozygosity | Chr 2: 5  Chr 8: 2 |
| Total autosomal indel calls | 1,569 |
| Novel or Rare Indels in regions of shared  homozygosity | Chr 2: 7  Chr 8: 0 |

**Table S3: List of Novel or Rare SNVs or Indels in regions of shared homozygosity**

| Genomic pos (hg19) | Reference sequence | Genotype | Variant Type | Gene Name | GeneID | Substitution |
| --- | --- | --- | --- | --- | --- | --- |
| Chr2:214,204,890 | AGCT | GCT/GCT | Amino acid deletion | SPAG16 | NM_024532 | K180- |
| Chr2:214,204,896 | AG | G/G | Deletion frameshift | SPAG16 | NM_024532 | R182nfs |
| Chr2:214,204,905 | GCT | A/A | Deletion frameshift | SPAG16 | NM_024532 | L185nfs |
| Chr2:217,124,179 | T | CC | Nonsynonymous | MARCH4 | NM_020814 | Q363R |
| Chr2:219,127,574 | G | CC | Nonsynonymous | GPBAR1 | NM_001077191 | R43P |
| Chr2:219,127,577 | G | CC | Nonsynonymous | GPBAR1 | NM_001077191 | R44P |
| Chr2:219,374,728 | G | AA | Nonsynonymous | USP37 | NM_020935 | S333F |
| Chr2:220,096,748 | AACC | A/A | amino acid deletion | ANKZF1 | NM_018089 | H83- |
| Chr2:220,345,397 | GT | G/G | Deletion frameshift | SPEG | NM_005876 | P1782nfs |
| Chr2:220,355,615 | CAG | C/C | Deletion frameshift | SPEG | NM_005876 | A3107nfs |
| Chr2:220,432,785 | G | +T | insertion frameshift | OBSL1 | NM_015311 | T424nfs |
| Chr8:70,744,822 | T | CC | Nonsynonymous | SLCO5A1 | NM_001146008 | E29G |
| Chr8:104,427,408 | T | CC | Nonsynonymous | DCAF13 | NM_030780 | L64P |

**Figure S1.** SNP genotype for individuals 1-5 on chromosome 2 (a) and 8 (b) from the CNVPartition module of Illumina’s GenomeStudio software. The region of minimal overlap of homozygosity shared by all three foetuses (individuals 3, 4, 5) is shown in shaded region. In each horizontal track, heterozygous SNP genotype calls appear as a horizontal row halfway between the homozygous genotypes (top and bottom of each track). Genomic coordinates of each region are presented in Supplementary Table 1. The entire length of each chromosome is presented from the end of the p arm (left) to the end of the q arm (right).

**a.**


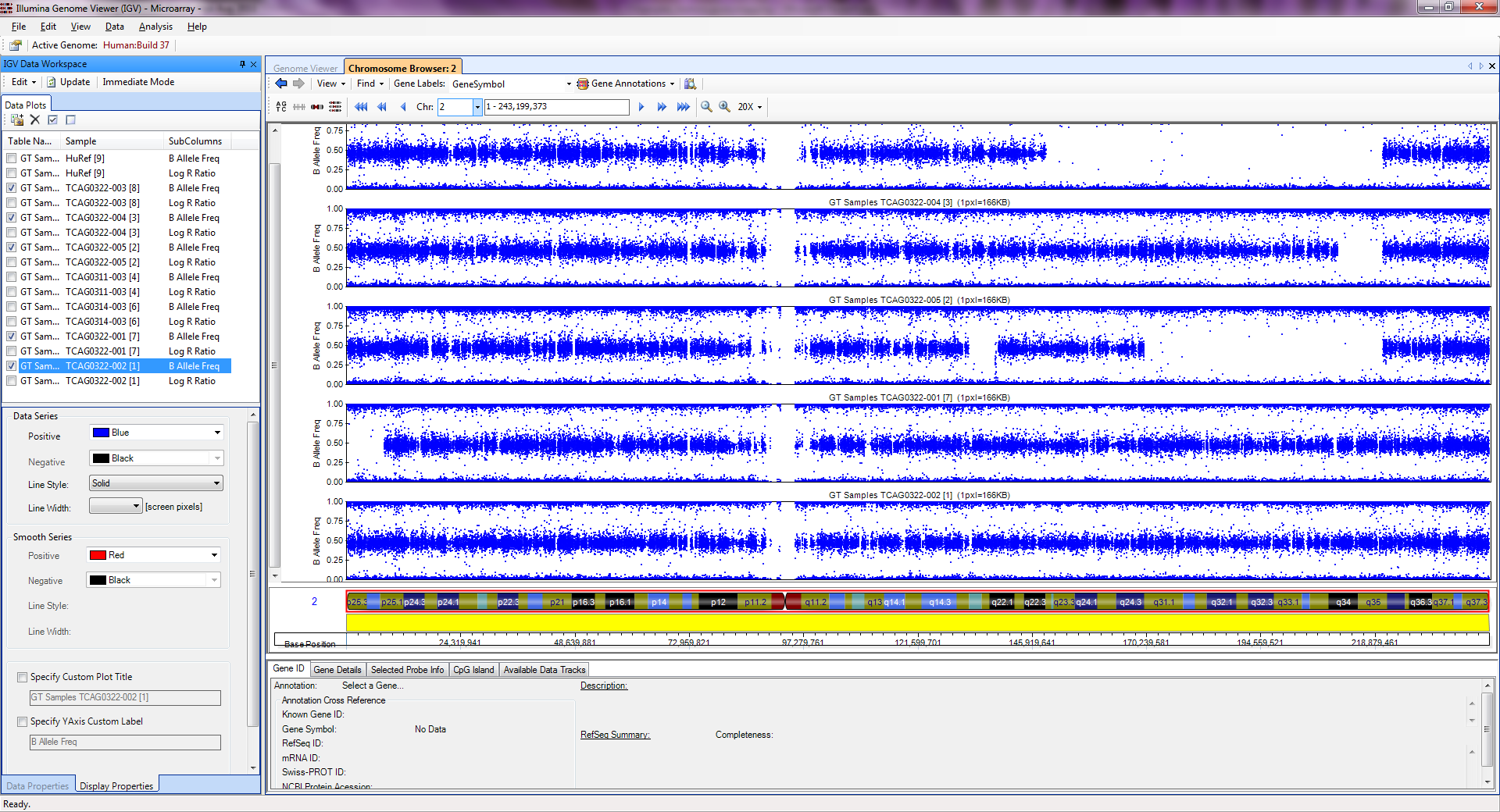


2

1

3

4

5

**b.**


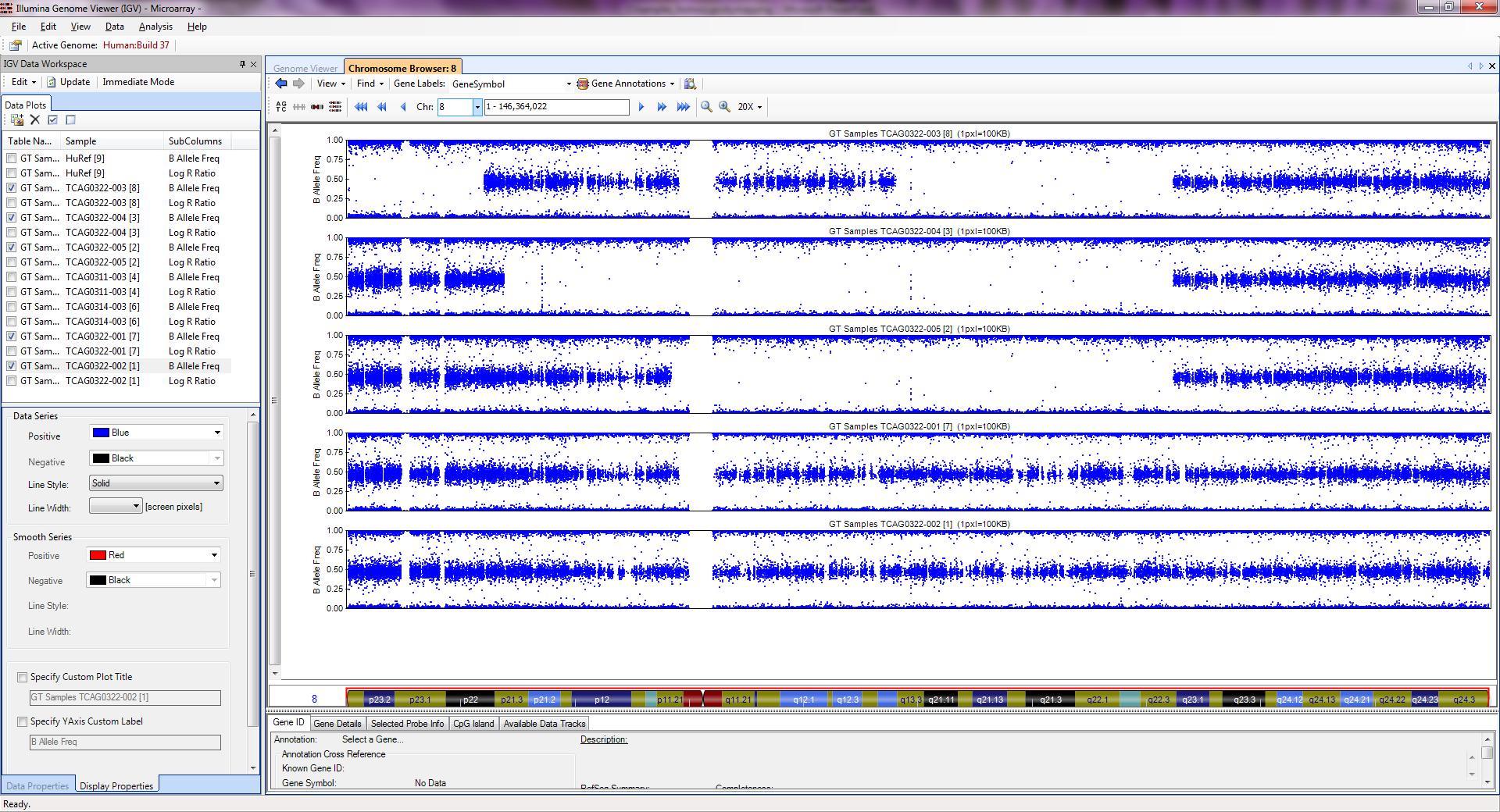


2

1

3

4

5
